# Immunotherapy Using Oxygenated Water and Tumor-Derived Exosomes Potentiates Antitumor Immune Response and Attenuates Malignancy Tendency in Mice Model of Breast Cancer

**DOI:** 10.1155/2021/5529484

**Published:** 2021-05-28

**Authors:** Nafiseh Pakravan, Ardeshir Abbasi, Zuhair Mohammad Hassan

**Affiliations:** ^1^Division of Immunology, Medical School, Alborz University of Medical Sciences, Karaj, Iran; ^2^Department of Immunology, Faculty of Medical Sciences, Tarbiat Modares University, Tehran, Iran

## Abstract

Breast cancer is one of the most common type of tumor and the leading cause of death in the world's female population. Various therapeutic approaches have been used to treat tumors but have not led to complete recovery and have even damaged normal cells in the body. Moreover, metastatic tumors such as breast cancer are much more resistant to treatment, and current treatments have not been very successful in treating them and remain a challenge. Therefore, new approaches should be applied to overcome this problem. Given the importance of hypoxia in tumor survival, we aimed to test the antitumor effects of oxygenated water to decrease hypoxia along with tumor-derived exosomes to target tumor. The purpose of administering oxygenated water and tumor exosomes was to reduce hypoxia and establish an effective immune response against tumor antigens, respectively. For this purpose, the breast cancer mice model was induced using the 4T1 cell line in Balb/c mice and treated with oxygenated water via an intratumoral (IT) and/or intraperitoneal (IP) route and/or exosome (TEX). Oxygenation via the IT+IP route was more efficient than oxygenation via the IT or IP route. The efficiency of oxygenation via the two routes along with TEX led to the best therapeutic outcome. Antitumor immune responses directed by TEX became optimized when systemic (IP) and local (IT) oxygenation was applied compared to administration of TEX alone. Results demonstrated a significant reduction in tumor size and the highest levels of IFN-*γ* and IL-17 and the lowest levels of IL-4 FoxP3, HIF-1*α*, VEGF, MMP-2, and MMP-9 in the IT+IP+TEX-treated group. Oxygenated water on the one hand could reduce tumor size, hypoxia, angiogenesis, and metastasis in the tumor microenvironment and on the other hand increases the effective immune response against the tumor systemically. This therapeutic approach is proposed as a new strategy for devising vaccines in a personalized approach.

## 1. Introduction

Hypoxia is an important feature of solid tumors. Several mechanisms are presumed to be involved in the development of hypoxic conditions within the tumor foci. They include limited perfusion and/or delivery of O_2_. Tumor cells adapt to hypoxia, persist in harsh conditions, and become more invasive and metastatic [1–4]. Normal cells typically die in hypoxic conditions while tumor cells adapt to a hostile hypoxic microenvironment and remain viable due to hypoxia-mediated proteomic and genomic changes within tumor cells [5, 6]. In addition, the selection pressure when subjected to hypoxia leads to the survival of more malignant subpopulations of tumor cells expressing MMP-2, MMP-9, and VEGF [7–9]. Hypoxic conditions within the tumor microenvironment lead to increased angiogenesis and subsequent resistance to treatment with a number of anticancer agents [10]. Various antiangiogenic therapies have not yet succeeded in treating tumors, and based on the prediction made by mathematical models, complete treatment based on angiogenesis inhibition is not possible [11, 12]. Accordingly, normalization of vasculature but not antiangiogenic therapy has also been proposed. This would help in restoring tissue architecture and establishing a normal level of oxygen in the damaged tissue [13].

Tumor cell resistance resulting from hypoxic conditions is the main obstacle preventing effective cancer treatment. Several approaches have been studied to facilitate cancer immunotherapy. Despite more than 40 years of effort, however, none of the approaches have been successful up to now. Given the important role of oxygen deprivation in tumor development, adaptation, and metastasis, it has been proposed that oxygenation can restore health by destroying cancer cells. Supporters of oxygen therapy claim that low levels of oxygen enable tumor cells to adapt and thrive. Accordingly, oxygenation of tumor cells interferes with their proteomic and genomic changes and destroys them [14–16]. In addition, the hypoxic condition within tumors inhibits antitumor immune responses [17–19].

Immunotherapeutic approaches focusing on tumor antigens are required to target the tumor itself, would have lower side effects, and are more effective than nonspecific approaches, such as chemotherapy or radiation therapy [20, 21]. In this regard, various approaches have been developed, one of which is the use of tumor-derived exosomes. Tumor-derived exosomes are microvesicles containing tumor antigens that can be uptaken by phagocytes. They are capable of interacting with T cells and affect T cell activation status [22].

Breast cancer is the most prevalent cancer and leading cause of death of women in the world [23]. On this basis and with the aim of modulating the tumor microenvironment and stimulating tumor antigen-specific immune responses, oxygenated water and tumor-derived exosomes were applied in a mice model of breast cancer in this study.

## 2. Materials and Methods

### 2.1. Ethics Statement

This study was commenced after obtaining the ethics code from the Ethical Committee of the Deputy of Alborz University of Medical Sciences under reference No: IR.ABZUMS.ERC.1397.154. The people involved in the project did their best to adhere to medical research ethical standards in all stages of the study.

### 2.2. Animals

Female BALB/c mice at the age of 8-10 weeks were purchased from the Rouyan Institute, Karaj, Iran. Given free access to food and water, mice were housed for one week before experiments and maintained in good standard conditions. All experiments were performed according to the Animal Care and Use Protocol of Alborz University of Medical Sciences.

### 2.3. Cell Line and Preparation of Mice Breast Cancer Model

The mouse breast cancer cell line 4T1 was obtained from the Pasture Institute, Cell Bank of Iran (NCBI, Tehran, Iran). The cells were cultured in RPMI 1640 medium supplemented with 10% fetal bovine serum (FBS), 1% glutamine (Thermo Fisher Scientific), 100 IU/ml streptomycin, and 100 IU/ml penicillin at 37°C in a humidified atmosphere.

A suspension of 0.15 ml containing 6 × 10^5^ 4T1 cells was injected subcutaneously in the dorsal flank regions [24]. The cages were coded, neoplastic masses were measured with a caliper, tumor size was measured every other day for all mice, and growth curves were prepared. Tumor volume was measured using the following formula:

(1) $$\begin{array}{c} \text{Tumor volume}=\frac{\left(\text{length}\times {\text{width}}^{2}\right)}{2}. \end{array}$$

### 2.4. Preparation and Exosome Isolation

Exosome was isolated from 4T1 cells cultured in RPMI 1640 medium supplemented with 10% exosome-free FBS which was prepared by centrifugation of FBS for 15 hours at 120,000 g and 4°C. Exosome was isolated from the culture media using gradient ultracentrifugation. Briefly, culture media was collected from 4T1 cells cultured for 72 h and centrifuged at 300 g for10 min to discard nonattached cells and cell debris. The supernatant was then passed through gradient ultracentrifugation (45 Ti Rotor, Beckman Coulter) at 2,000 g for 10 min, 30,000 g for 10 min, and 110,000 g for 70 min to eliminate any conceivable apoptotic bodies, macrovesicles, and contaminating proteins, respectively.

### 2.5. Characterization of Exosome Using DLS, SEM, TEM, and Western Blot Analysis

Exosome pellets were diluted in 200 *μ*l. After that, the size, homogeneity, and concentration of the isolated exosome were checked and determined using a Zetasizer Nano ZS90 (Malvern Instruments Ltd., Worcestershire, UK) and the Pierce Bicinchoninic Acid (BCA) Protein Assay Kit (Thermo Fisher Scientific, Waltham, MA), respectively. Moreover, the purity and morphology of the exosome obtained was assured using scanning electron microscopy (SEM) and transmission electron microscopy (TEM).

Exosome surface markers were also identified using Western blot. Following denaturation, protein separation was carried out using sodium dodecyl sulfate polyacrylamide gel electrophoresis (SDS-PAGE). Following the transfer of the samples onto polyvinylidene difluoride (PVDF) membranes, the expression of exosome-specific marker proteins was examined using antibodies against tumor susceptibility gene 101 (TSG101), CD63, CD81, and CD9. Data analysis was performed using ImageJ software (version 1.50i, National Institute of Health, Bethesda, MD, USA).

### 2.6. Treatment with/without Oxygenated Water with/without an Exosome

**Figure 1:**
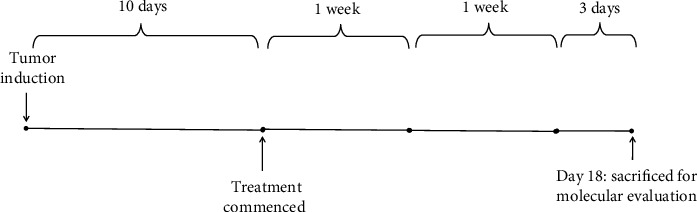
Protocol of treatment with oxygenated water and/or exosomes. Ten days after tumor implantation using the 4T1 cell line, the animals were treated with oxygenated water and/or exosomes. Treatment with oxygenated water was performed via the intratumoral and/or intraperitoneal route and was performed on a daily basis. Exosomes were administered via the subcutaneous route every three days. Three days after the last treatment, the animals were euthanized and immunological evaluations were performed.

Treatments were commenced on day 10 after tumor implantation when the tumor was palpable (≈75-100 mm^3^). To evaluate the therapeutic effect of oxygenated water and/or the exosome, six groups were considered (*n* = 6‐8) as described in Table 1 and treated based on the protocol shown in Figure 1. The treatment lasted for 15 days, and on the 18th day, three days after the last injection, the spleen and tumor tissues of the mice were isolated. Tumor volume was measured every other day with a caliper for 15 days. The measurement was performed by one person twice. Oxygenated water was administered via an intraperitoneal (IP) and/or intratumoral (IT) route and a tumor-derived exosome (TEX) was administered via the subcutaneous (SC) route. Oxygenated water containing 85 ppm oxygen (OXAB Co., Gorgan, Iran) was injected daily [25] and TEX (100 *μ*g) were injected into the area around the tumor every three days.

### 2.7. Tissue Preparation for Immunohistochemical Staining

After completion of the treatment protocol, the animals were anesthetized using a ketamine and xylazine mixture and the spleen was taken out. The dissected spleens were fixed in neutral buffered 10% formalin. This was followed by paraffin embedding, and 5 *μ*m thick sections were prepared on a rotary microtome (Leica, Germany). The sections were placed on polylysine-coated slides and used for immunohistochemical staining [26]. The tissue sections were blocked with 0.3% Triton X-100 and 10% goat serum in PBS (pH 7.3) for 30 min. Then, primary antibodies were added and incubated overnight at room temperature including interferon-*γ* (IFN-*γ*, orb10878, Biorbyt, Cambridge, UK), interleukin-17 (IL-17, orb48920, Biorbyt, Cambridge, UK), IL-4 (orb318722, Biorbyt, Cambridge, UK), and Forkhead box P3 (orb156940, FoxP3, Biorbyt, Cambridge, UK). After washing with 0.01 M PBS, the tissue sections were incubated with FITC-conjugated donkey anti-rabbit IgG (Biorbyt, Cambridge, UK) diluted in 0.01 M PBS (1 : 200) as the secondary antibody for 2 h at room temperature. Then, after rinsing with 0.01 M PBS, the sections were stuck to glass slides and observed using a fluorescence microscope. DAPI (4′-6-diamidino-2-phenylindole) was used for nuclei staining in each section. Quantification and analysis of the immunohistochemically stained tissue sections were performed after taking digitized images using a Zeiss Axioplan 2 fluorescent microscope. Image J software (version: 1.52 h) was used to analyze the digitized images by an observer blinded to the origin of the sample.

### 2.8. RNA Extraction and Real-Time PCR

Total RNA was extracted from frozen tumor using the TRIzol™ Reagent (Invitrogen) according to a standard protocol and a previous report [27]. NanoDrop 2000c (Eppendorf, Germany) was used to determine the quality and quantity of RNA concentrations. Expression of mRNA for hypoxanthine phosphoribosyl transferase (HPRT), hypoxia inducible factor-1*α* (HIF-1*α*), vascular endothelial growth factor receptor-A, and matrix metalloproteinase- (MMP-) 2 and 9 was determined using the ABI StepOnePlus thermocycler (Applied Biosystems, Sequence Detection Systems, Foster City, CA) and the SYBR® Green PCR Master Mix (Applied Biosystems, Life Technologies, Paisley, United Kingdom) according to the manufacturer's instructions. Each reaction contained 10 *μ*l Master Mix, 1 *μ*l (100 nM) primers for HPRT, HIF-1*α*, VEGF-A, and MMP-2, 9, and 1 *μ*l (200 ng) template cDNA synthesized with cDNA kits (Parstous, Tehran, Iran) and 8 *μ*l diethyl pyrocarbonate (DEPC) water. The sequences for primers were forward 5′-CAGGACTGAAAGACTTGCTC-3′ and reverse 5′-AGGTCAGCAAAGAACTTATAGC-3′ for HPRT, forward 5′-GGATCAAACCTCACCAAAGC-3′ and reverse 5′-GCAGGAACATTTACACGTCTG-3′ for VEGF-A, forward 5′-ACAGGACAGTACAGGATGC-3′ and reverse 5′-GGGAGAAAATCAAGTCGTGC-3′ for HIF-1*α*, forward 5′-GGACAAGAACCAGATCACATAC-3′ and reverse 5′-CGTCGCTCCATACTTTTAAGG-3′ for MMP-2, and forward 5′-GTGTCTGGAGATTCGACTTG-3′ and reverse 5′-CCTTGTTCACCTCATTTTGG-3′ for MMP-9. The primers' efficiency and specificity, the fidelity of real-time PCR, and melting curve analysis were determined as before [27]. Thermocycler conditions included an initial step at 95°C for 10 min, followed by 40 cycles at 95°C for 10 sec, 56-63°C for 30 sec (the annealing temperature of each primer), and 72°C for 30 sec. The HPRT gene was chosen as the internal control against which mRNA expression of the target gene was normalized. The resultant gene expression level was presented as 2^−ΔΔCt^, in which ΔCt was the difference between Ct values of the target gene and the reference gene [27].

### 2.9. Statistical Analysis

Statistical operations were performed using GraphPad Prism software (GraphPad Software, San Diego, CA) to analyze the data using one-way ANOVA to compare between groups followed by the Tukey post hoc test. The results of tumor size were analyzed using two-way ANOVA and the Bonferroni post hoc procedure. Differences were considered statistically significant when the *p* value was less than 0.05.

## 3. Results

### 3.1. Evaluation of Exosomes

**Figure 2:**
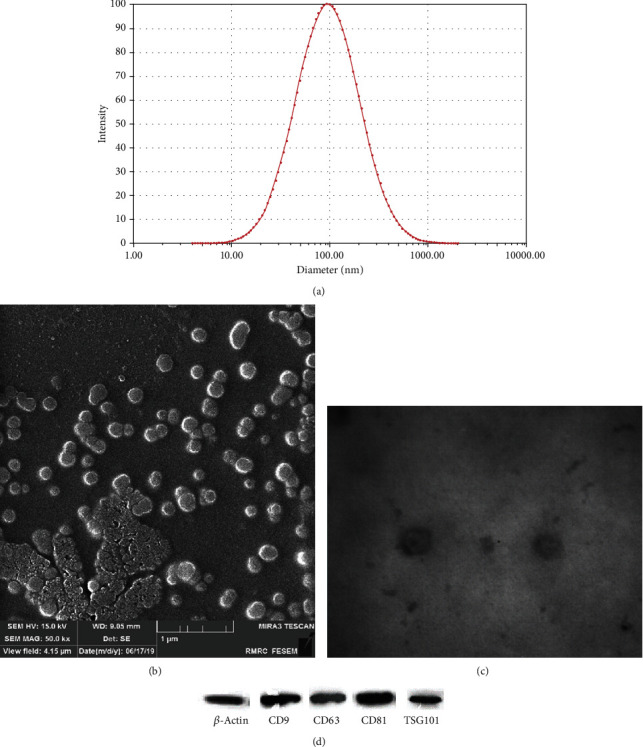
(a) Size distribution of 4T1-derived exosomes using Dynamic Light Scattering (DLS) which showed that the isolated exosomes were homogenous. Views of exosomes using (b) scanning electron microscopy (SEM) and (c) transmission electron microscopy (TEM). (d) Western blot analysis for detecting the expression of CD9, CD63, CD81, and TSG101 in exosomes derived from the 4T1 tumor cell line. *β*-Actin was used as control.

The exosomes derived from supernatants of the 4T1 cell culture were evaluated by dynamic light scattering (DLS), transmission electron microscopy (TEM), scanning electron microscopy (SEM), and Western blotting. As shown in Figure 2(a) and based on DLS, the isolated exosomes had a homogeneous size with a peak of 96 ± 8.5 SD nm which indicates the appropriate size range (30-120 nm) of the exosomes. Consistently, the results obtained by examining the morphology of the exosomes using electron microscopy confirmed the homogeneity of the population in the range of 30-120 nm, their spherical morphology, and a bilayer phospholipid membrane (Figures 2(b) and 2(c)). The results of Western blot analysis (Figure 2(d)) also showed that exosomes secreted from 4T1 tumor cells express CD81, CD9, CD63, and TSG101 markers along which *β*-actin was used as the internal control.

### 3.2. Tumor Size and Growth

**Figure 3:**
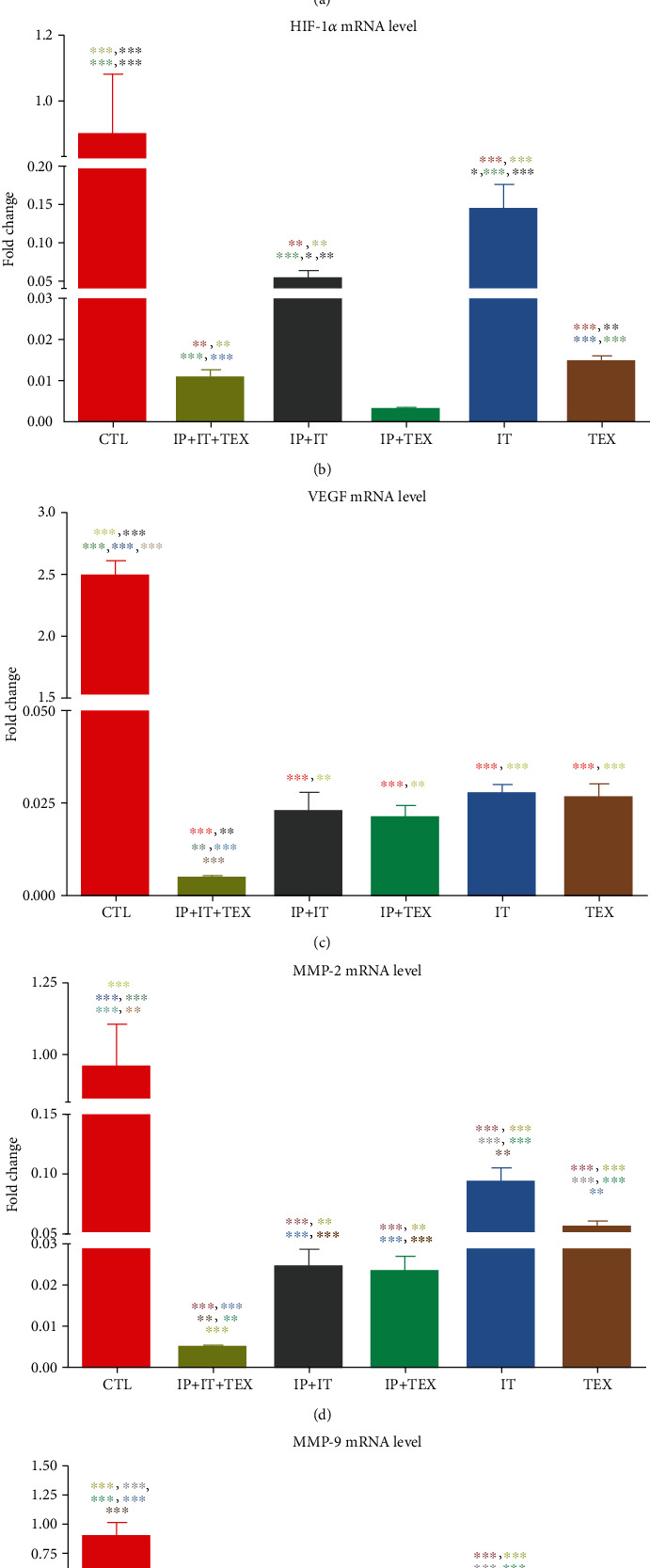
Comparison of tumor growth and mRNA expression of HIF-1*α*, VEGF-A, MMP-2, and MMP-9 in the tumor loci of control and treatment groups with oxygenated water and/or exosomes. Injection of oxygenated water via the intratumoral and/or intraperitoneal route was performed on a daily basis. Exosomes were administered via the subcutaneous route every three days. Tumor dimensions were measured using a caliper (vernier) every other day, and tumor size was calculated as described in TEX. Three days after the last injection, the animals were euthanized, tumor was isolated, RNA was extracted, and cDNA was synthesized. Real-time PCR using Cyber Green was performed and the quantification of each gene was normalized against HPRT as the reference gene. Significant difference *vs*. control according to line color (∗). Data are presented as mean ± SEM. TEX: tumor-derived exosome.

As shown in Figure 3(a), tumor size in the control group was increasingly growing, but it demonstrated a decreasing trend in the treatment groups. On day 13 after the beginning of treatment, there was a significant difference between the control group and the IT (*p* < 0.001), IT+IP (*p* < 0.05), IT+IP+TEX (*p* < 0.05), and TEX (*p* < 0.05) groups. In addition, there was a significant difference between the control group and the groups treated with IT+TEX (*p* < 0.0001), IT+IP (*p* < 0.0001), IT+IP+TEX (*p* < 0.0001), or TEX (*p* < 0.0001) on day 15 after the beginning of treatment. Tumor size was not significantly different among the groups under treatment.

### 3.3. Hypoxia, Angiogenesis, and Metastatic Potential of Tumor

The difference in mRNA expression of HIF-1*α*, VEGF-A, MMP-2, and MMP-9 genes at the tumor loci is shown in Figures 3(b)–3(e). As shown in Figure 3(b), the expression of the HIF-*α* gene in the control group was significantly higher than those in the groups under treatment (*p* < 0.001). However, the expression of the HIF-*α* gene in the TEX group was significantly higher than those in the IP+IT (*p* < 0.01), IP+IT+TEX (*p* < 0.001), IT+TEX (*p* < 0.001), and IT (*p* < 0.05) groups. In addition, the expression of the HIF-*α* gene in the IT group was significantly higher than those in the IP+IT+TEX, IT+TEX, and IP+IT groups (*p* < 0.001). It was also notable that the expression of the HIF-*α* gene in IT+TEX group was significantly more than those in the IP+IT+TEX and IP+IT groups (*p* < 0.001). On this basis, the expression of the HIF-*α* gene can be summarized as follows:

(2) $$\begin{array}{c} \text{IP}+\text{IT}+\text{TEX}<\text{IP}+\text{IT}<\text{IT}+\text{TEX}>\text{IT}<\text{TEX}<\text{CTL}. \end{array}$$

Examination of VEGF-A gene expression showed (Figure 3(c)) that the expression of VEGF-A in the control group was significantly higher than those in the groups under treatment (*p* < 0.001). However, there were significant differences among the groups under treatment with oxygenated water with or without exosome. The expression of the VEGF-A gene in the TEX group was significantly higher than those in the IT (*p* < 0.01), IT+TEX (*p* < 0.001), IT+IP (*p* < 0.001), and IP+IT+TEX groups (*p* < 0.001). Also, the IT group expressed significantly higher VEGF-A than the IT+TEX, IP+IT, and IP+IT+TEX groups (*p* < 0.001). As shown in Figure 3(c), the IT+TEX group also expressed more VEGF-A than the IP+IT (*p* < 0.01) and IP+IT+TEX groups (*p* < 0.001). The IP+IT and IP+IT+TEX groups were also statistically different in VEGF-A expression (*p* < 0.01). The expression of the VEGF gene can be summarized as follows:

(3) $$\begin{array}{c} \text{IP}+\text{IT}+\text{TEX}<\text{IP}+\text{IT}<\text{IT}+\text{TEX}<\text{IT}<\text{TEX}<\text{CTL}. \end{array}$$

To evaluate the metastatic potentials of a tumor, the expression of the MMP-2 gene was evaluated. As shown in Figure 3(d), the MMP-2 expression in the control group was significantly higher than those in the groups under treatment (*p* < 0.001). However, among the treated groups, the expression of the MMP-2 gene in the TEX group was significantly higher than those in the IT (*p* < 0.05), IT+TEX (*p* < 0.001), IP+IT (*p* < 0.001), and IP+IT+TEX groups (*p* < 0.001). Also, the IT group had a higher MMP-2 expression level than the IP+IT, IT+TEX, and IP+IT+TEX groups (*p* < 0.001). There was no statistical difference in the expression of MMP-2 between the IP+IT and IT+TEX groups. Finally, the expression level of MMP-2 in the IP+IT+TEX group was significantly lower than those in the IP+IT and IT+TEX groups (*p* < 0.001). The expression level of the MMP-2 gene can be summarized as follows:

(4) $$\begin{array}{c} \text{IP}+\text{IT}+\text{TEX}<\text{IP}+\text{IT}\approx \text{IT}+\text{TEX}<\text{TEX}<\text{IT}<\text{CTL}. \end{array}$$

Similar to MMP-2, the expression of the MMP-9 gene (Figure 3(e)) in the control group was significantly higher than those in the groups under treatment (*p* < 0.001). However, among the treatment groups, the expression of the MMP-9 gene in the TEX group was significantly higher than those in the other treatment groups (*p* < 0.001). Moreover, the IT group expressed a higher MMP-9 level than the IT+TEX (*p* < 0.05), IP+IT (*p* < 0.001), and IP+IT+TEX (*p* < 0.001) groups. Finally, the expression level of MMP-9 in the IT+TEX group was markedly higher than those in the IP+IT and IP+IT+TEX (*p* < 0.01) groups. There was no statistical difference in the level of MMP-9 expression between the IP+IT+TEX and IP+IT groups. The order of MMP-9 gene expression level can be summarized as follows:

(5) $$\begin{array}{c} \text{IP}+\text{IT}+\text{TEX}\approx \text{IP}+\text{IT}<\text{IT}+\text{TEX}<\text{IT}<\text{TEX}<\text{CTL}. \end{array}$$

### 3.4. Profile of T Cell Response

The spleen is considered as a lymphatic organ that acts locally (in response to antigens of blood origin) and systemically [28]. The role and importance of the spleen in regulating systemic immune responses is such that it is referred to as the barometer of systemic immune responses [29]. Measurement of immune responses in the spleen shows us the balance and amplitude of the immune responses throughout the body and the extent to which these responses may be involved to control tumor growth.

**Figure 4:**
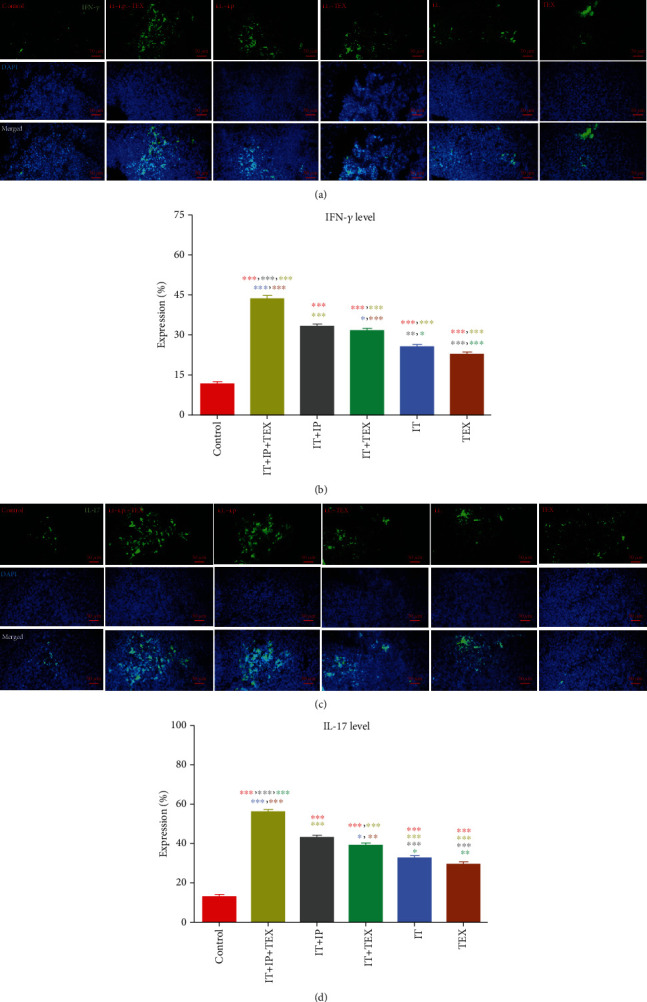
Effect of oxygenated water and/or exosome treatment on anti-tumor immune response. Expression levels of Th1 and Th17 markers including IFN-*γ* and IL-17 were evaluated in spleen tissue sections (a, c) by immunohistochemical staining of serial spleen sections using corresponding antibody and analyzed (b, d). The data are shown as the mean ± SEM.

On this basis, the levels of IFN-*γ*, IL-4, IL-17, and FoxP3, as representatives of Th1, Th2, Th17, and regulatory T cells (Treg) [26, 27], were determined in the spleen. As shown in Figures 4(a) and 4(b), IFN-*γ* expression in the spleen of the control group was significantly lower than those of the groups under treatment (*p* < 0.001). However, IFN-*γ* expression levels also significantly differed between the treatment groups. The amount of IFN-*γ* expression in the treated groups can be expressed as follows:

(6) $$\begin{array}{c} \text{CTL}<\text{TEX}<\text{IT}<\text{IT}+\text{TEX}\approx \text{IP}+\text{IT}<\text{IP}+\text{IT}+\text{TEX}. \end{array}$$

Accordingly, the level of IL-17, which like IFN-*γ* is an inflammatory cytokine, was significantly lower in the spleen of the control group than in the treatment groups (*p* < 0.001). As illustrated in Figures 4(b) and 4(c), both Th1 and Th17 responses, which are indicators of inflammatory responses, were significantly increased in the treatment groups compared to the control group. Similar to the trend observed for IFN-*γ*, IL-17 levels in the treated groups can be expressed as follows:

(7) $$\begin{array}{c} \text{CTL}<\text{TEX}<\text{IT}<\text{IT}+\text{TEX}\approx \text{IP}+\text{IT}<\text{IP}+\text{IT}+\text{TEX}. \end{array}$$

**Figure 5:**
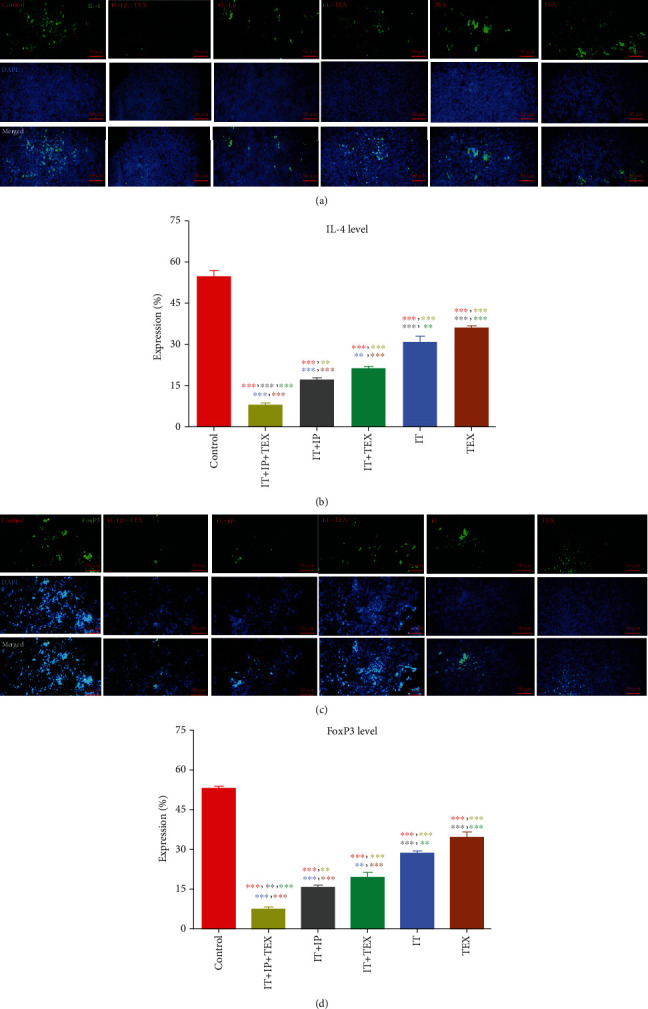
Impact of treatment with oxygenated water and/or exosome on tumor-promoting immune responses. Two markers of Treg and Th2 including FoxP3 and IL-4, respectively, were evaluated. Microscopic view of immunohistochemical staining of serial sections in spleen tissue sections using anti-FoxP3 and -IL-4 antibodies is shown (a, c). The percentage of each molecule in the spleen was analyzed (b, d). The data are shown as the mean ± SEM.

In contrast, IL-4 was significantly increased in the spleen of the control group compared to the treatment groups (*p* < 0.001, Figures 5(a) and 5(b)). Furthermore, the level of IL-4 significantly differed among the treatment groups. On this basis, the order of IL-4 was different from the case of IFN-*γ* and IL-17 as indicated below:

(8) $$\begin{array}{c} \text{IP}+\text{IT}+\text{TEX}<\text{IP}+\text{IT}\approx \text{IT}+\text{TEX}<\text{IT}<\text{TEX}<\text{CTL}. \end{array}$$

FoxP3 levels were also evaluated as an indicator of Treg in the spleen. As shown in Figures 5(c) and 5(d), the level of Treg in the spleen of the control group was significantly higher than those in the treatment groups (*p* < 0.001). Notably, the level of Treg also varied among the treatment groups. As expected, the FoxP3 level order was in a trend different from that of IFN-*γ* and IL-17 and similar to IL-4 in the control and treatment groups as follows:

(9) $$\begin{array}{c} \text{IP}+\text{IT}+\text{TEX}<\text{IP}+\text{IT}\approx \text{IT}+\text{TEX}<\text{IT}<\text{TEX}<\text{CTL}. \end{array}$$

## 4. Discussion

Tumor hypoxia is a severe problem and has detrimental effects on tumor therapy because it reduces the sensitivity of chemotherapy and radiation therapy. Given the important role of oxygen deprivation in tumor development, adaptation, and metastasis, it has been proposed that oxygenation can restore health by destroying cancer cells. Supporters of oxygen therapy claim that low levels of oxygen enable tumor cells to adapt and thrive. Accordingly, oxygenation of tumor cells interferes with their proteomic and genomic changes and can destroy them [14, 15]. The initial idea of oxygen therapy for cancer treatment using ozone or hydrogen peroxide, decomposing into oxygen, dates back decades ago [30–37]. Later studies have also shown beneficial effects of oxygen therapy in some cancers [9, 38–41]. For example, it has been shown that the effect of chemotherapy is significantly increased immediately after hyperbaric oxygen therapy [42]. Other investigations indicated that oxygen potently affects regulatory immune responses which are dominant within the tumor loci [17, 18]. Indeed, hypoxia promotes Treg recruitment to the site of tumor, enhances their activity in the tumor foci, and suppresses antitumor effector T cell response [19]. Accordingly, previous reports suggested oxygen-mediated angiogenesis [43–45], vascular normalization by proper oxygenation [46], and antimetastatic effects of hyperbaric oxygen [47–49] in contrast to hypoxia [48–51]. More importantly, a previous study demonstrated that drinking of oxygenated water containing 30-120 ppm oxygen has no harmful effect on whole blood count or on the liver enzymes and increased the Th1/Th2 ratio [25]. Clinical and experimental studies demonstrated that oxygenated water has physiological and immunological benefits and supplies the body with more oxygen [52–55]. In pathological conditions, there are some clinical reports describing the beneficial effects of drinking oxygenated water for patients suffering from a large variety of diseases, such as head and neck carcinomas [25, 52, 56]. Ozone or hydrogen peroxide has been proposed to increase oxygen and decrease hypoxia. Both ozone and hydrogen peroxide have shown antitumor effects. Administration of ozonated water was shown to increase intratumoral blood perfusion and improve hypoxic conditions within tumor loci increasing the therapeutic outcome. Hydrogen peroxide could also increase apoptosis and decrease proliferation and multiphase cell cycle arrest in 4T1 cells to some extent [57, 58]. A previous report applying oxygen nanobubble water on a similar mice model of breast cancer led to a 25% reduction in tumor volume [59]. Beneficial effects of oxygenation have been attributed to the involvement of oxygen in the activation of the p53 tumor suppressor gene [60] and clearance of lactate which is thought to be an important oncometabolite in the metabolic reprogramming of tumors [61]. Lactate helps acidification of the tumor microenvironment which in turn favors processes such as angiogenesis and metastasis. More importantly, lactate also causes immunosuppression which is an indication of a worse clinical prognosis [62]. This study also demonstrated the inhibitory effects of intratumoral and/or intraperitoneal administration of oxygenated water on angiogenesis, represented by VEGF-A, and metastatic potentials, represented by MMP-2 and MMP-9 levels. On this basis, the beneficial effects of oxygenation are not restricted to the hyperbaric or drinking form of oxygen. Not surprisingly, oxygen has also been mentioned as an adjuvant or vitamin O [47, 63–66]. Considering tumor as a systemic disease with local manifestation [67, 68], oxygenation via intratumoral plus intraperitoneal routes was more efficient than oxygenation via either an intratumoral or intraperitoneal route. The intraperitoneal route is regarded as a systemic route [69] and systemic oxygenation seems to fight systemic tumors favoring immune responses. In parallel, oxygenation via an intratumoral route also fights hypoxic conditions and tumors protecting local immune responses. However, a desirable therapeutic outcome using oxygen and tumor-derived exosomes was achieved in this study as discussed below.

Oxygenation alone is not sufficient to combat tumors. Indeed, oxygenation just turns an immunosuppressive condition into an immunopermissive microenvironment and decreases extracellular adenosine-mediated tumor protection [70, 71]. On this basis, stimulation of antitumor immune cells has been proposed to be required for tumor eradication. To devise a double-edged immunotherapeutic approach which modulates the tumor microenvironment and stimulates and potentiates an antitumor immune response, exosomes as a source of tumor antigens were also applied along with oxygenated water in this study. Utilization of tumor-derived exosomes for immunotherapy of cancer has been proposed and investigated before [72]. In the case of the 4T1 mice model of breast cancer, immunotherapy using exosomes in a previous study could just partially slow down tumor growth by almost half but administration of exosomes along with oxygenated water in this study decreased tumor size by 1/8th [73]. Another *in vitro* study demonstrated that 4T1 cell-derived exosomes help in the maturation of dendritic cells [74]. The results of this study indicated that the efficiency of oxygenation along with exosomes gave rise to a better therapeutic outcome. Molecular evaluations suggest that oxygenation via two routes, namely, intratumoral and intraperitoneal, led to a desirable therapeutic outcome when applied with exosomes. Based on the results of this study, therefore, antitumor immune responses directed by exosomes were more potent and optimized when systemic (IP) and local (IT) oxygenation was applied as compared with administration of exosomes alone. This was also evidenced by a similar efficiency between the two groups under treatment with IP+IT or IT+TEX. The importance of oxygenation became evident by the lowest therapeutic efficiency in the TEX group compared with the other groups under treatment.

Although we did not evaluate macrophage phenotype, VEGF-A, MMP-2, and MMP-9 levels reflect tumor microenvironment changes as they are produced by M2 macrophages [75, 76]. As for the importance of modulating the tumor microenvironment by oxygenated water, it is notable that exosomes derived from tumor resident macrophages caused tumor growth and progression [77]. In addition, hypoxia educates and promotes macrophages toward the M2 phenotype which favors tumor growth [78]. Accordingly, 4T1 cell-derived exosomes can somewhat stimulate macrophages to produce proinflammatory cytokines [79] consistent with another study demonstrating the polarization of macrophages toward M1 activation status [80]. Given tumor immunometabolism, polarization of macrophages to the M1 phenotype promotes tumor vessel normalization, tumor reoxygenation, and metastasis inhibition as a result of metabolic competition that oxygenation makes among immune and tumor cells [81, 82]. Consistently, there are reports indicating the importance of the intratumoral oxygen gradient and oxygen availability as a major regulator of M1↔M2 phenotype transition [83, 84]. Nevertheless, utilization of exosomes along with oxygenated water in this study was also important to potentiate antitumor immune response, as one previous study reported that oxygenation alone failed to change the macrophage phenotype acquired in the tumor hypoxic condition [85].

## 5. Conclusion

This study proposes systemic and local oxygenation along with immunotherapy using tumor-derived exosomes as a new approach of combination immunotherapy. This approach stimulates an antitumor immune response using systemic oxygenation along with tumor-derived exosomes, and simultaneously, local oxygenation modulates the tumor microenvironment, weakens the tumor-protecting immune mechanism, and empowers and lets antitumor immune cells enter the tumor loci.

## Figures and Tables

**Table 1:** Animal grouping and treatments.

	Group					
Treatment	1	2	3	4	5	6
Oxygenated water	-	(IT)∗	-	(IT)∗	(IP+IT) ∗	(IP+IT)^∗^
Exosome (TEX)	—	—	(S.C.)∗	(S.C.)∗	—	(S.C.) ∗

^∗^IP: intraperitoneal; IT: intratumoral; S.C.: subcutaneous; TEX: tumor-derived exosome.

## Data Availability

The data that support the findings of this study are available from the corresponding author, Dr. Nafiseh Pakravan, upon reasonable request.
